# Quinine controls body weight gain without affecting food intake in male C57BL6 mice

**DOI:** 10.1186/1472-6793-13-5

**Published:** 2013-02-08

**Authors:** Philippe Cettour-Rose, Carole Bezençon, Christian Darimont, Johannes le Coutre, Sami Damak

**Affiliations:** 1 Nestlé Research Center, Vers-chez-les-Blanc, Lausanne 1000, Switzerland; 2 Organization for Interdisciplinary Research Projects, The University of Tokyo, Tokyo, Japan

**Keywords:** Obesity, Food intake, Fat, Body composition, Gastrointestinal tract

## Abstract

**Background:**

Quinine is a natural molecule commonly used as a flavouring agent in tonic water. Diet supplementation with quinine leads to decreased body weight and food intake in rats. Quinine is an *in vitro* inhibitor of Trpm5, a cation channel expressed in taste bud cells, the gastrointestinal tract and pancreas. The objective of this work is to determine the effect of diet supplementation with quinine on body weight and body composition in male mice, to investigate its mechanism of action, and whether the effect is mediated through Trpm5.

**Results:**

Compared with mice consuming AIN, a regular balanced diet, mice consuming AIN diet supplemented with 0.1% quinine gained less weight (2.89 ± 0.30 g vs 5.39 ± 0.50 g) and less fat mass (2.22 ± 0.26 g vs 4.33 ± 0.43 g) after 13 weeks of diet, and had lower blood glucose and plasma triglycerides. There was no difference in food intake between the mice consuming quinine supplemented diet and those consuming control diet. Trpm5 knockout mice gained less fat mass than wild-type mice. There was a trend for a diet-genotype interaction for body weight and body weight gain, with the effect of quinine less pronounced in the Trpm5 KO than in the WT background. Faecal weight, energy and lipid contents were higher in quinine fed mice compared to regular AIN fed mice and in Trpm5 KO mice compared to wild type mice.

**Conclusion:**

Quinine contributes to weight control in male C57BL6 mice without affecting food intake. A partial contribution of Trpm5 to quinine dependent body weight control is suggested.

## Background

Quinine is a natural molecule extracted from the bark of the cinchona tree commonly used as a flavoring agent in tonic water and bitter lemon and, at higher doses, for the treatment of some forms of malaria. Consumption of quinine by rats strongly reduces food intake and body weight [1-7]. The decrease in food intake was initially attributed to the intense bitter taste of quinine but it was later shown that diminished food intake is observed with rats consuming a diet supplemented with quinine, but not with a diet supplemented with iso-bitter sucrose octaacetate, suggesting that palatability is not the only cause of decreased food intake in rats consuming quinine in the diet [1]. Furthermore it is unclear from the rat experiments whether there is a direct effect of quinine on body weight, independently of food intake. Quinine was recently shown to inhibit the activity of Trpm5 [8] a calcium activated cation channel expressed in the taste buds [9], gastrointestinal tract [10], pancreas [11] and other hollow organs, involved in taste signaling and glucose homeostasis. Here we present evidence that quinine controls body weight independently of food intake in male C57BL6 mice and investigate its mechanism of action, including a possible role of Trpm5 in mediating the effect of quinine on body weight control.

## Results

Initially we planned to use encapsulated quinine to mask its bitterness and eliminate any potential impact on food intake caused by unpalatable diet. During the course of optimising the encapsulation procedure, we found that C57BL6 mice consume equal amounts of regular diet or non-encapsulated quinine supplemented diet (data not shown). Therefore the encapsulation approach was dropped and all subsequent experiments were carried out with regular, non encapsulated quinine.

### Quinine fed mice gain less body weight and fat mass than mice on a regular diet (study 1)

**Figure 1:**
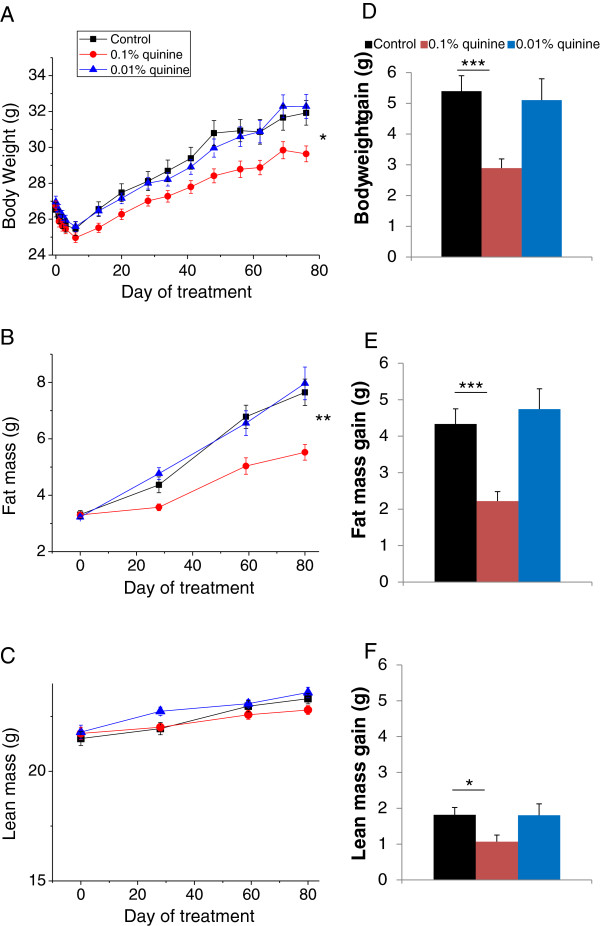
Body weight, fat and lean mass of WT mice on control diet, diet + 0.1% quinine, or diet + 0.01% quinine, showing lower body weight and fat mass gains with 0.1% quinine but not with 0.01% quinine, compared to regular diet. * p < 0.05, **p < 0.01, ***p < 0.001.

We supplemented a regular balanced diet (AIN) of wild type male mice with quinine, and measured their body weight and fat mass for 13 weeks. We tested quinine concentrations of 0.01%, which is the highest concentration allowed in drinks for human consumption, and 0.1%. Compared with wild type mice consuming regular diet (WT control), wild-type mice consuming 0.1% quinine supplemented diet (WT quinine) had lower body weight (p < 0.05, Figure 1A) and lower fat mass (p < 0.01) (Figure 1B), whereas there was no significant difference in lean mass between WT control and WT quinine (Figure 1C). WT quinine mice gained less weight (2.89 ± 0.30 g vs 5.39 ± 0.50 g p < 0.001, Figure 1D), less fat mass (2.22 ± 0.26 g vs 4.33 ± 0.43 g p < 0.001, Figure 1E) and less lean mass (1.07 ± 0.18 g vs 1.82 ± 0.20 g p < 0.05) than WT control mice. There was no significant difference in body weight, fat mass or lean mass between WT control and WT mice consuming 0.01% quinine (Figure 1).

### Food intake is not different between quinine fed mice and mice fed a regular diet (study 1)

**Figure 2:**
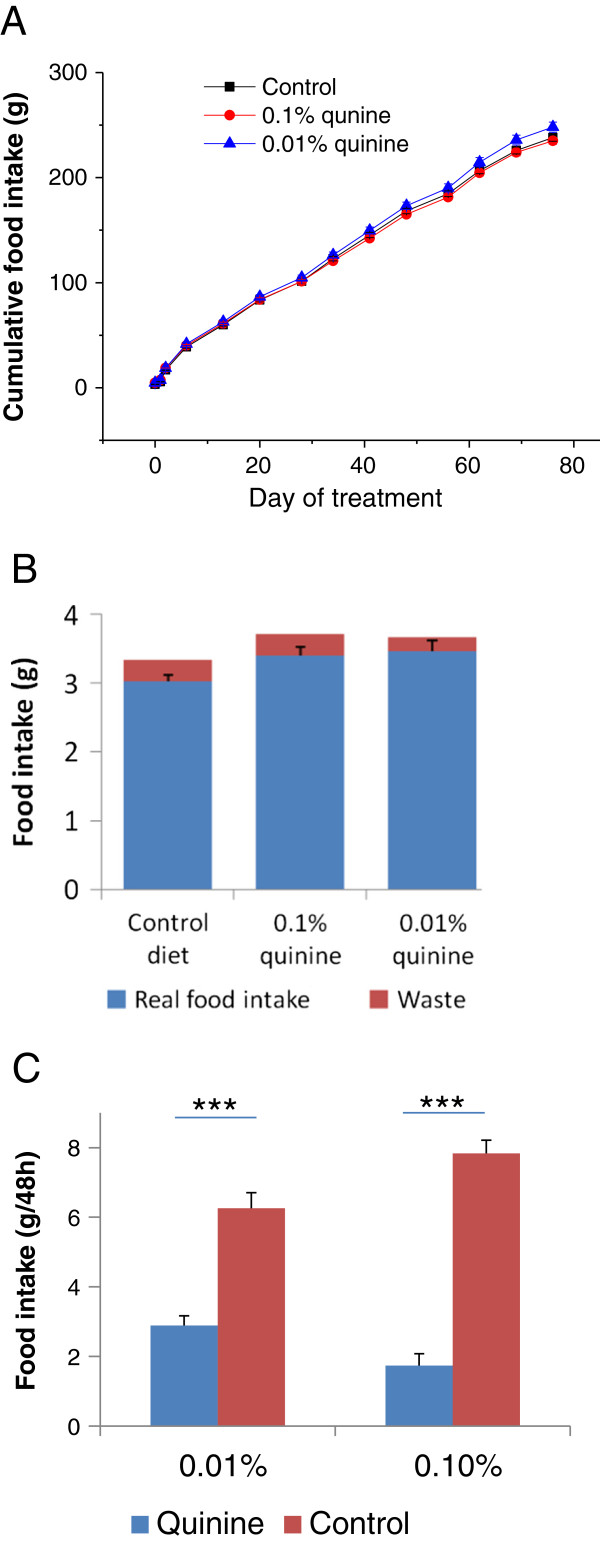
**Food intake of WT mice on AIN diet with or without quinine. A**. Cumulative food intake of mice on control AIN diet, AIN diet + 0.1% quinine, or AIN diet + 0.01 % quinine, showing no difference between any of the groups. **B**. Daily real food intake and food waste of wild type mice consuming AIN diet, or AIN diet supplemented with 0.1% or 0.01% quinine. Real food intake was calculated by subtracting waste from measured food intake. There is no significant difference between groups with different diets. There is a trend for increased real food intake by animals fed quinine supplemented diets. **C**. Diet preference tests, comparing AIN diet and AIN diet supplemented with either 0.01% or 0.1% quinine, showing aversion for the quinine supplemented diets.

There was no significant difference in cumulative food intake between the WT control mice, WT quinine mice, and mice fed a diet supplemented with 0.01% quinine (238 ± 3.5 g, 235 ± 3.2 g, 248 ± 4.8 g, respectively, Figure 2A). A preliminary experiment showed that the percentage of waste was very small, and real daily food intake (measured food intake minus waste) was not significantly different between groups, although there was a trend for increased real food intake by animals fed quinine supplemented diets (Figure 2B). When given the choice between two diets with or without quinine (Study 5), wild type mice avoid the quinine containing diet in favour of the regular diet, demonstrating aversion for the bitter taste of quinine (Figure 1C). In contrast, when given only one choice, mice consume the same amounts of regular or quinine supplemented diet (Figure 2B). Thus the decreased body weight and fat mass gains observed in the WT quinine mice are not caused by diminished intake of unpalatable food.

### Metabolic parameters are improved by treatment with quinine (study 1)

**Figure 3:**
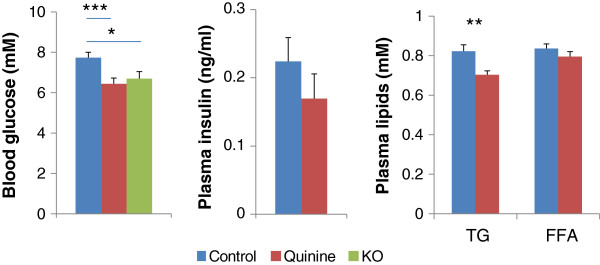
**Blood glucose, plasma insulin, triglycerides (TG), and free fatty acids (FFA) from 0.1% quinine fed (quinine) and control diet fed (control) WT mice, and from Trpm5 KO mice fed a control diet (KO) for blood glucose. **Blood glucose is more elevated in the control than in quinine or Trpm5 KO mice. There is no difference in plasma insulin between control and quinine. TG, but not FFA are more elevated in control than in quinine. * p < 0.05, **p < 0.01, ***p < 0.001.

Compared with WT control mice, blood glucose and plasma triglycerides were lower in WT quinine mice (p < 0.005, and p < 0.01, respectively, Figure 3). Blood glucose was lower in Trpm5 KO mice than in control (Study 2, p < 0.05). There was no significant difference in plasma free fatty acids and insulin between WT control and WT quinine mice (Figure 3).

### Effect of diet supplementation with 0.1% quinine on body weight and body composition of wild type and Trpm5 knockout mice (study 2)

**Figure 4:**
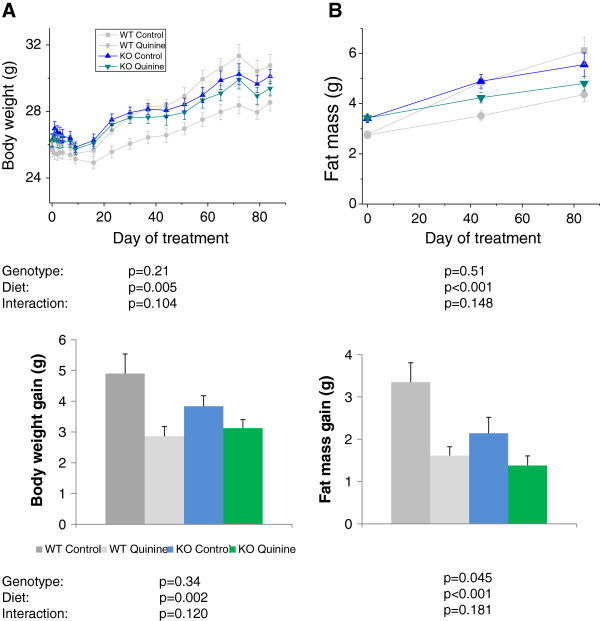
**Body weight and fat mass of WT and Trpm5 knockout mice on control diet or diet + 0.1% quinine. **The p values for the effects of diet, genotype and the interaction are shown below each graph. Body weight and fat mass gains are significantly diminished by quinine. Fat mass gain is significantly lower in Trpm5 KO mice. There is a trend for interaction diet*genotype for body weight, were the effect of quinine is larger in the WT than in the knockout background.

Since quinine inhibits Trpm5 *in vitro*, in this experiment we investigated whether the effect of quinine on body weight and body composition was mediated through Trpm5. To this effect, we measured body weight and composition of WT control, WT quinine, Trpm5 KO mice with regular AIN diet (KO control) and Trpm5 KO mice with 0.1% quinine supplemented AIN diet (KO quinine) and compared the effect of genotype (WT vs KO), diet (control vs quinine) and genotype*diet interaction. The means for body weight gain and fat mass gain for each group are given in Table 1. There was a significant effect of diet on body weight repeated measurements and gain (control > quinine, p < 0.01), but no effect of genotype. There was a significant effect of diet on fat mass repeated measurements and gain (Control > quinine, p < 0.001). There was a significant effect of genotype on fat mass gain (WT > KO, p < 0.05). There was a trend for an interaction diet*genotype for repeated measurements of body weight (p = 0.10) and body weight gain (p = 0.12) (Figure 4). These data confirm the effect of quinine on body weight and fat mass found in study 1. Trpm5 KO mice gain less fat mass than WT animals. The diet*genotype interaction trend suggests that part of the effect of quinine on body weight and fat mass may be Trpm5-dependent. There was no significant effect of genotype or diet on lean mass gain (Values in Table 1).

**Table 1:** Results of study 2, showing means ± SEM for gains in body weight, fat mass, lean mass and body weight after quinine removal from the diet in the four experimental groups of mice

	**Body weight gain (g)**	**Fat mass gain (g)**	**Lean mass gain (g)**	**Body weight gain after quinine removal (g)**
WT control	4.90 ± 0.63	3.35 ± 0.46	1.19 ± 0.14	2.14 ± 0.22
WT quinine	2.86 ± 0.31	1.61 ± 0.21	0.66 ± 0.19	2.00 ± 0.28
KO control	3.84 ± 0.33	2.19 ± 0.38	1.13 ± 0.30	1.39 ± 0.36
KO quinine	3.12 ± 0.28	1.38 ± 0.23	1.38 ± 0.31	1.78 ± 0.24

### Mice do not regain weight after removal of quinine (study 2)

After 13 weeks of consuming a diet supplemented with quinine, mice were switched to a control diet for 4 weeks. During that period there was no significant effect on weight gain of diet and a strong trend for genotype (p = 0.087) with no significant interaction (values in Table 1). Thus quinine treated mice do not regain weight after being switched to regular diet, whereas Trpm5 KO mice tend to gain less weight than WT mice.

### Effect of quinine and Trpm5 KO on energy balance (study 2)

**Figure 5:**
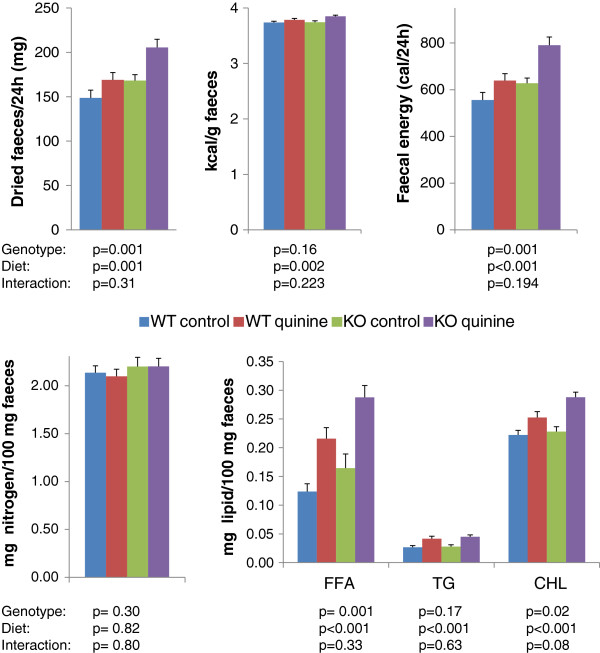
**Faecal weight, and calorie, nitrogen and lipid contents of faeces from WT and Trpm5 KO mice consuming a regular diet or a 0.1% quinine supplemented diet. **The p values for the effects of diet, genotype and the diet*genotype interaction are shown below each graph. FFA: free fatty acids, TG: triglycerides, CHL: cholesterol.

Faeces from WT control, WT quinine, KO control and KO quinine mice were collected and their total energy, fat and protein contents were measured (Figure 5). The amount of dried faeces per 24 h, faecal energy per gram of faeces, faecal energy per 24 h, faecal free fatty acids, faecal triglycerides, and faecal cholesterol were higher in mice receiving the quinine supplemented diet than those on control diet (Figure 5). The amount of dried faeces per 24 h, faecal energy per 24 h, faecal free fatty acids, and faecal cholesterol were higher in Trpm5 KO mice than in control mice (Figure 5). Phospholipids were not detectable in the faeces. There was no significant effect of genotype or diet on faecal nitrogen content. There was no genotype*diet interaction for any of those parameters.

### The effect of quinine on fat mass gain is small and short lived in diet-induced obese mice fed a high fat diet (study 3)

**Figure 6:**
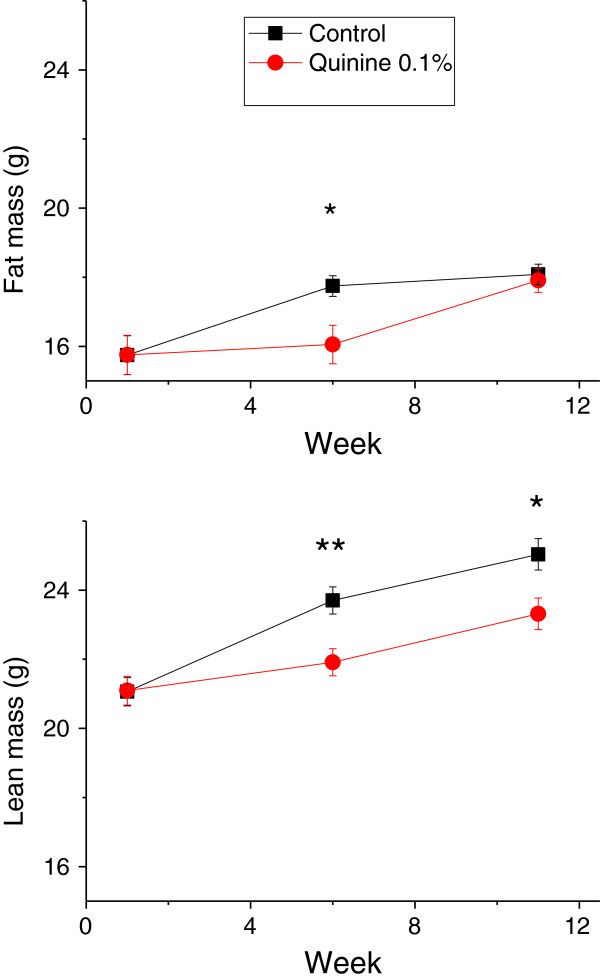
**Body weight and body composition of obese wild type mice receiving control or 0.1% quinine supplemented high fat diet. **Measures were taken at the beginning, 6 weeks and 11 weeks after the onset of the study. At 6 weeks after onset of the experiment, both lean and fat mass are lower in the quinine treated group, whereas at week 11 only lean mass is lower. * p < 0.05, **p < 0.01.

The aim of this experiment was to determine if supplementation of the diet of obese mice with quinine would lead to weight loss. Diet-induced obese mice were fed a 45% fat diet with or without supplementation with 0.1% quinine for 12 weeks. WT quinine mice gained less weight than WT control mice, but fat mass was lower in WT quinine mice only after 6 weeks of exposure to the experimental diet (p < 0.05, Figure 6). At week 11, there was no significant difference in fat mass. Lean mass was lower in quinine-treated mice at week 6 and week 11 (p < 0.01 and p < 0.05, respectively). Cumulative food intake was lower in mice fed quinine supplemented high fat diet than in control (177 ± 2.4 g vs 189 ± 3.1 g p < 0.05). Thus when high fat diet is used, the effect of quinine on fat mass is small, short lived and caused at least in part by diminished food intake.

### Twenty four-hour energy expenditure and activity are not different between WT control, WT quinine, and KO control mice (study 4)

**Figure 7:**
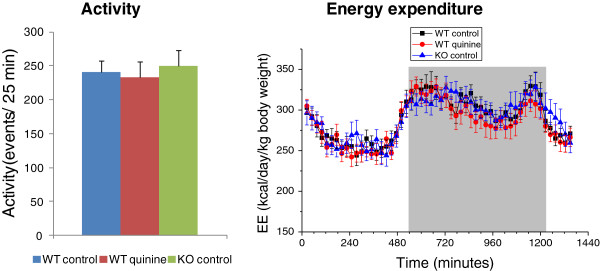
**Activity and energy enpenditure. **Twenty-four hour activity shown as mean events per 25 minute period, and energy expenditure of AIN-fed WT control, WT quinine and KO control mice show no difference between conditions. For energy expenditure, the dark period is represented by a grey box.

Twenty four-hour energy expenditure, respiratory quotient and activity were measured after mice were fed the experimental diets for two weeks and did not differ between WT control, WT quinine and KO control fed AIN (Figure 7) or high fat diet (not shown).

### Markers of hepatic toxicity and of systemic inflammation are normal in quinine-fed mice (study 1)

To rule out the possibility that the diminished body weight gain observed in the quinine fed animals was caused by liver toxicity or general inflammation, we measured plasma liver enzymes and cytokines. Plasma aspartate-amino-transferase (AST) and alanine-amino-transferase (ALT), which are elevated in case of hepatic toxicity, were within normal range in mice fed a diet containing 0.1% quinine and not significantly different from control (AST: 47.2 ± 1.5 U/L and 43.2 ± 1 U/L; ALT 20.9 ± 1.6 U/L and 18.2 ± 0.7 U/L for control and quinine, respectively).

**Figure 8:**
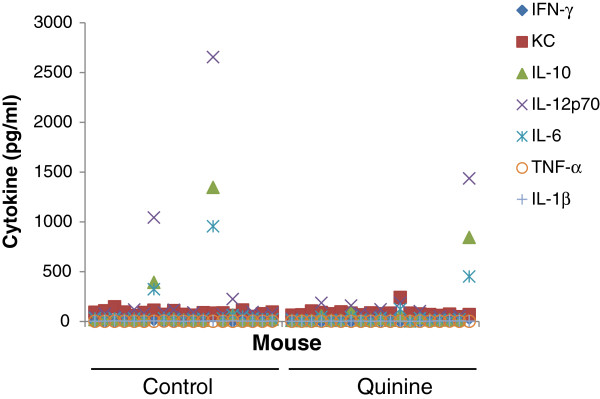
**Inflamation markers. **Plasma cytokine concentrations in individual WT mice fed a diet containing 0.1% quinine or a control diet showing elevated levels of cytokines in two control mice and one quinine fed mouse, but no difference in the levels of cytokines between diets.

Two mice in the control group and one mouse in the quinine fed group had elevated INF-γ, IL-10, IL-12p70, and IL-6, suggesting that those three mice had systemic inflammation (Figure 8). For all other mice the levels of IL-1β, IL-12p70, INF-γ, IL-6, and TNF-α were either undetectable, in most cases, or slightly above the detection threshold. The levels of KC and IL-10 were detectable above threshold in all mice, with no significant difference between control and quinine (medians for control and quinine, respectively, KC: 86 pg/ml and 74 pg/ml; IL-10: 19 pg/ml and 13 pg/ml).

Together these data show that there is no evidence of liver toxicity or systemic inflammation associated with quinine supplemented diet.

## Discussion

In this study, we tested the effect of quinine on mouse body weight and body composition and investigated possible mechanisms, including the role of Trpm5. Mice consuming a balanced diet supplemented with 0.1% quinine gained less fat mass than mice on a regular diet and maintained this difference at least for one month after quinine was removed from the diet.

Previous experiments investigating the role of quinine in food intake and body weight were conducted using rats [1-7]. Quinine has an intense bitter taste to humans and is aversive to rodents. Adding quinine to the diet of rats results in a large decrease in food intake accompanied by diminished body weight. The decrease in food intake is observed with rats consuming a diet supplemented with quinine, but not with a diet supplemented with iso-bitter sucrose octaacetate, suggesting that palatability is not the only cause of decreased food intake in rats consuming quinine in the diet [1]. Our results differ from those obtained with rats in that food intake was not diminished in mice consuming quinine supplemented diet compared to those consuming regular diet. In preliminary experiments we found that when given the choice between two diets with or without 0.1% quinine, wild type mice avoid the quinine containing diet in favour of the regular diet, demonstrating aversion for the bitter taste of quinine. In contrast, when given only one choice, mice consume the same amounts of regular or quinine supplemented diet. Food intake was carefully measured in studies 1–4 and the results were consistent for all the studies where AIN diet was used. We also measured waste in a separate experiment and found that it was minimal, and real food intake was not affected by addition of quinine. Thus, whereas decreased food intake explains part of the quinine induced weight loss in rats, it does not in regular diet fed mice. Rats are more likely to decrease their food intake because of palatability than mice.

Smaller gain in body weight and fat mass may be caused by increased energy expenditure, due to increased metabolism or activity, or decreased nutrient ingestion and/or absorption. We found an increase of total faeces, faecal energy and lipid content in the quinine treated mice compared to mice on control diet. There was no difference in protein content. Faecal carbohydrate content cannot be directly measured since sugars are converted in the colon by the gut microflora into short chain fatty acids [12]. The largest contribution to faecal lipid difference between quinine treated and control mice was from free fatty acids, suggesting that absorption of fatty acids, not digestion of triglycerides is deregulated by quinine treatment. The 24 h faecal weight is larger in quinine fed mice than in mice fed regular diet. The difference (~20 mg) cannot be accounted for by the difference in lipid content, as the difference in faecal FFA between quinine fed mice and control diet fed mice is only ~0.1 mg. The bulk of the difference in faecal weight most likely results from differences in the amount of undigested starch. Indirect calorimetry and measurement of activity showed no difference between WT control, WT quinine and KO control. Altogether these data suggest that quinine administration deregulates intestinal nutrient absorption rather than energy expenditure. All mice, including quinine fed animals, looked healthy without any signs of distress during the experiment. There were no biological signs of liver toxicity, and biological signs of systemic inflammation were found in two mice in the control group and one mouse fed quinine. Thus, it is unlikely that insults to visceral organs may have accounted for the diminished weight gain observed in quinine fed mice.

Quinine-fed mice did not regain weight during a 4-week follow up period after quinine-containing diet was replaced with a regular AIN diet. This fact is of clinical relevance. If quinine were to be investigated as a body weight control measure in humans, it may not be necessary to provide it on a continuous basis.

Is the effect of quinine on body weight and body composition Trpm5 dependent? For body weight and body weight gain there is a trend for an interaction genotype-diet, with a clear effect of quinine on WT type and a very small non significant effect on the knockout. This trend suggests the existence of a Trpm5 and quinine-dependent mechanism of body weight and fat mass gain prevention. Total faeces, caloric and lipid faecal contents are increased by quinine treatment both in the KO and WT backgrounds (no interaction diet-genotype) indicating that they represent Trpm5-independent quinine-dependent mechanisms of body weight control. Quinine is likely to act on multiple targets, including possibly Trpm5, bitter taste receptors [13], which are expressed in the gastrointestinal tract [14], potassium channels [15,16] or by direct activation of G-proteins [17].

What is then the Trpm5-dependent mechanism of diminished body weight gain? Trpm5 is expressed in the taste buds [9], gastrointestinal tract [10], pancreas [11] and other hollow organs. In the gastrointestinal tract Trpm5 is expressed in solitary cells disseminated throughout the gut, some of which also co-express T1rs, the receptors for sweet and *umami* tastes, suggesting that they might be chemosensory cells. Intestinal Trpm5 expressing cells produce endogenous opioids (β-endorphin and Met-enkephalin) and uroguanylin, and they secrete β-endorphin in response to various stimuli, particularly hypertonic stimuli, in a Trpm5-dependent fashion [18]. Opioids are well known to inhibit intestinal motility. It is tempting to speculate that inactivation of Trpm5 by gene knockout leads to increased intestinal motility through diminished release of β-endorphin thereby reducing intestinal nutrient uptake. Consistent with this hypothesis increased faecal weight, and faecal caloric and lipid contents are found in Trpm5 KO mice compared to wild type mice, although the differences are less marked as with quinine versus control. Given that the Trpm5-dependent prevention of weight gain takes place over a very long period, it is understandable that its underlying physiological changes are subtle. Quinine, on the other hand decreases gastrointestinal transit [19] and therefore would increase faecal weight, energy and lipids through a mechanism independent of Trpm5 inhibition.

Quinine mediated control of body weight and fat mass is clearly observed in mice fed a balanced diet (AIN) but is less clear when mice are fed high fat diet and interpretation of the results is confounded by lower food intake in the quinine group. Mice fed high fat diet overeat because of the palatability of fat, and this overeating may have been smaller in the quinine group because of bitterness.

## Conclusion

Our data show that quinine contributes to the control of body weight and fat mass without impacting food intake in male mice fed a balanced diet and therefore may constitute a novel tool for the fight against the obesity epidemic.

## Methods

### Animals

All experiments were conducted according to Swiss animal experimentation laws and guidelines and were approved by an internal animal experimentation ethics committee and by the Veterinary Office of the Canton de Vaud. Mice were maintained at 22 degrees C with a 12 h dark-12 h light cycle.

### Study design

#### Study 1: body weight and body composition of wild-type mice consuming quinine supplemented diet

Wild type C57BL6/J male mice three months old at the beginning of the experiment were used. They were fed a balanced semi synthetic diet (AIN 93 G, 64% calories from carbohydrates, 20% from proteins, 16% from fat, Diet # D10012G, Research Diets, New Brunswick, NJ, USA) with or without different doses of quinine, for 13 weeks. Three groups of 20 mice each were studied: A. Wild type fed AIN 93 G diet; B. Wild type fed AIN 93 G diet supplemented with 0.1% quinine HCl; C. Wild type fed AIN 93 G diet supplemented with 0.01% quinine HCl. 0.01% quinine corresponds to the maximal concentration allowed in drinks for human consumption. Groups were matched for fat mass assessed by NMR at the beginning of the study.

Body weight, food and fluid intake were measured weekly throughout the study. Body composition was measured at the beginning of the study, and on week 4, 8 and 12. At the end of week 13, mice were fasted for 6 hours, a drop of blood was collected by making a small incision in the tail vein, from which blood glucose was measured, then the mice were anesthetised with 3% isoflorane and blood was collected from the abdominal aorta for measurement of plasma insulin, aspartate-amino-transferase (AST), alanine-amino-transferase (ALT), pro-inflammatory cytokines, free fatty acids and triglycerides.

#### Study 2: body weight and body composition of WT and Trpm5 KO mice consuming quinine supplemented diet

The aims of this study were to: 1. confirming the results of Study 1; 2. determine if the effect of quinine on body weight was mediated by Trpm5; 3. Investigate the effect of quinine on energy intake; 4. Determine if mice regain weight once quinine is removed from the diet.

Wild type and Trpm5 KO male mice three months old at the beginning of the experiment were used. Trpm5 knockout (KO) mice (obtained from Deltagen, San Mateo, CA, USA) were described in [20]. The KO mice were backcrossed for six generations into C57BL6/J background. Mice were fed a balanced semi synthetic diet (AIN 93 G) with or without 0.1% quinine for 13 weeks. In order to determine if the effect on body weight persists when the mice are no longer fed quinine, all mice were fed AIN 93 G without quinine after the initial 13-week trial for an additional 4 weeks. Four groups of 15 male mice each were studied: A. Wild type C57BL6/J fed AIN 93 G diet (WT control); B. Wild type C57BL6/J fed AIN 93 G diet supplemented with 0.1% quinine HCl (WT quinine); C. Trpm5 KO fed AIN 93 G diet (KO control); D. Trpm5 KO fed AIN 93 G diet supplemented with 0.1% quinine HCl (KO quinine). Groups A and C were matched with groups B and D, respectively, for fat mass and body weight at the beginning of the study.

Body weight, food and fluid intake were measured weekly throughout the study. Body composition was measured at the beginning of the study, on week 7 and on week 13. On weeks 1, 7 and 13, mice were placed on a grid above a piece of cardboard covering the bottom of the cage for 72 hours. The faeces were separated from food crumbs and powder, and collected daily for measurement of weight, macronutrient content and direct calorimetry. On week 13 blood glucose was measured from a drop of blood collected by making a small incision in the tail vein.

#### Study 3: body weight and body composition of wild-type obese mice consuming quinine supplemented high fat diet

The aim of this experiment was to determine if supplementation with quinine of the diet of obese mice would lead to weight loss. Seven-week old wild type male C57BL6/J mice were fed a diet with 60% calories from fat (research diets D12492) for 9 weeks to make them obese. They were then fed a 45% high fat diet (research Diets D12451) for one week in order to maintain their weight and to get habituated to the 45% fat diet. Then one group of 19 mice continued to be fed 45% high fat diet (control), and the other group of 19 mice was fed 45% high fat diet containing 0.1% quinine-HCl for 12 weeks. Groups were matched for fat mass assessed by NMR at the beginning of the study.

Body weight, food and fluid intake were measured weekly throughout the study. Body composition was measured at the beginning of the study, and on week 6 and 11.

#### Study 4: energy expenditure and activity

Three groups of 10 male mice 3 month old and weight matched, were tested: A. Wild type mice without quinine, B. Wild type mice with 0.1% quinine HCl, C. Trpm5 KO mice without quinine.

Mice were fed a balanced diet (AIN-93 G) with or without 0.1% quinine HCl for two weeks, then were fed high fat diet (60% energy from fat, Research Diets #12492) with or without 0.1% quinine HCl for two weeks. Energy expenditure was measured after each two-week period; the mice were placed into metabolic cages for a 24-hour acclimation period then V0_2_, VC0_2_, respiratory quotient (RQ) and activity were measured over 24 hours using an Oxylet system (Panlab, Barcelona, Spain) with O_2_ and CO_2_ sensors, coupled to a SEDACOM infrared system to measure activity. Respiratory quotient (RQ) is the ratio of carbon dioxide production to oxygen consumption. Energy expenditure was calculated using the Weir equation (EE = 1.44 x VO_2_ x (3.815 + 1.23 x RQ)).

#### Study 5: two diet preference tests

Two groups of 8 male 3-month old C57BL6/J mice were tested. Every mouse received two diets, AIN-93 G and AIN-93 G supplemented with 0.01% quinine HCl (Group A) or with 0.1% quinine HCl (Group B). The two diets were placed on the cage lid, separated by the water bottle. The positions of the diets were switched after 24 hours. The diets were weighed at the beginning of the experiment, and 24 h and 48 hours later. The amount consumed from each diet were calculated and compared by paired Student T-tests.

### Body composition

Body composition was determined in duplicate using an EchoMRI-900 Body Composition Analyzer (Echo Medical System, LLC, Houston, TX, USA).

### Food intake

Food intake was calculated by weighing the diet and subtracting the weight at the end of the measured period from that at the beginning. Spillage of food on the bottom of the cage was collected and weighed to ensure the quality of food intake measurements.

In a preliminary experiment, we measured food waste by wild-type C57BL6/J mice consuming AIN diet, AIN diet + 0.1% quinine HCl or AIN diet +0.01% quinine HCl, 7 male mice each. Mice were placed on a grid above a piece of cardboard covering the bottom of the cage for 72 h. Food powder and pellet crumbs were separated from faeces and weighed.

### Analysis of faecal contents

Mice were placed on a grid for 72 h and faeces were collected every 24 hours at the bottom of the cages which were covered with a piece of absorbent cardboard to minimise contamination of the faeces by urine. Faeces collected during a 24 h period were vacuum oven-dried (50°C, 24 hours) and weighed.

For measurement of caloric contents, the dried material was turned into powder with a mortar. The powder was compacted into two pellets using a Pellet Press 2811 (Parr Instrument Company, Moline, IL, USA). The dried materials were added to 0.4 g benzoic acid and burned in a 6100 Oxygen Bomb Calorimeter (Parr Instrument Company) to measure their caloric content. Measurements were done in duplicate.

For measurement of nitrogen content, 2 mg of dried and homogenised faeces were placed in a sealed capsule and inserted into an elemental analyser (CHNS-932, Leco, St Joseph, MI, USA) and combusted at 1000°C in presence of oxygen. The total contents of N in the sample were quantitatively converted to N_2_ and subsequently determined by measurement of the thermal conductivity after separation of the gaseous components. Measurements were done in quadruplicate.

Faecal lipids were measured as described [21]. Briefly, lipids were extracted from 100 mg of dried faeces with 2 ml chloroform-methanol 2:1. The organic phase was recovered and 1 ml of water was added to it. Following a second centrifugation, the organic phase was recovered and evaporated under N_2_ for 30 min and resuspended in 500 μl 1% Triton X 100 in Chloroform, evaporated under N_2_ for 10 min and resuspended in 500 μl water. The following commercial kits were used for the measurement of lipids, according to the manufacturer’s protocol: NEFA HR (2), Wako, Osaka, Japan, for free fatty acids; TG PAP 150, BioMerieux, Marcy l’Etoile, France for triglycerides; LabAssay Cholesterol, Wako, for cholesterol, LabAssay Phospholipids, Wako, for phospholipids.

### Blood parameters

Mice were food deprived for six hours, then glucose was measured from a drop of blood obtained from an incision of the tail vein, using a glucometer (Ascensia Elite, Bayer, Germany). The measures were done in duplicate.

After measurement of glucose, the mice were anesthetised with 3% isoflorane and blood was collected from the aorta for the remaining measurements using commercial kits (NEFA HR (2), Wako, for free fatty acids; TG PAP 150, BioMerieux, Marcy l’Etoile, France for triglycerides; Ultra Sensitive Mouse Insulin ELISA Kit, Crystal Chem, Downers Grove, IL, USA, for insulin, and Roche reagents, Meylan, France for ALT and AST) according to the manufacturer’s protocol. Cytokines were measured using a multiplex immunoassay (Meso Scale Discovery, Gaithersburg, MD, USA). The cytokines included in the multiplex assay are Interleukin (IL)-1β, IL-12p70, Interferon-γ (INF-γ), IL-6, keratinocyte chemoattractant (KC), IL-10, and Tumour Necrosis Factor-α (TNF-α)

### Statistical analysis

#### Studies 1, 3 and 4

For all parameters except body weight, fat mass and lean mass, the data were analysed with the General Linear Model univariate of the statistics program SPSS with the measured parameter as a within-subject factor and diet as a between subject factor. When more than two groups were compared and a significant difference was found, a *post hoc* Tukey test was performed to determine which groups differ.

For body weight, fat mass and lean mass, the data were analyzed with the General Linear Model Repeated Measures of the statistics program SPSS with the measured parameter as a within-subject factor and diet as a between subject factor. When more than two groups were compared and a significant difference was found, a *post hoc* Tukey test was performed to determine which groups differ.

For each cytokine the non-parametric Mann Whitney test was used to assess the difference between control and quinine fed mice and the results are presented as medians.

#### Study 2

The data were analyzed with the General Linear Model of the statistics program SPSS with the measured parameter as a within-subject factor and diet and genotype as between subject factors. The interaction diet*genotype was also analyzed.

Data are presented as mean ± SEM. A p value <0.05 was considered significant.

## Competing interests

All of the authors are, or were, employees of Nestec Ltd, which is a subsidiary of Nestlé Ltd. and provides professional assistance, research, and consulting services for food, dietary, dietetic, and pharmaceutical products of interest to Nestlé Ltd. No other conflicts of interest are reported. The study was funded by Nestec Ltd.

## Authors’ contribution

The authors’ responsibilities were as follows: CD, JleC, SD: designed the study; PCR and CB: organized and executed the trials; SD, CD and JleC: interpreted the data, wrote and edited the manuscript. All authors red and approved the final manuscript.
